# Cognitive, motor, and behavioral outcomes in preterm infants exposed to opioids

**DOI:** 10.1038/s41390-025-04048-3

**Published:** 2025-04-12

**Authors:** Philipp Steinbauer, Julia Kühnl, Karin Pichler, Sophie Stummer, Katrin Klebermass-Schrehof, Philipp Deindl, Claudia Lindtner, Monika Olischar, Sophia Brandstetter, Renate Fuiko, Angelika Berger, Vito Giordano

**Affiliations:** 1 Division of Neonatology, Pediatric Intensive Care and Neuropediatrics, Department of Pediatrics, Comprehensive Center for Pediatrics (CCP), Medical University of Vienna, Vienna, Austria; 2 Department of Neonatology and Pediatric Intensive Care Medicine, University Children’s Hospital, University Medical Center Hamburg-Eppendorf, Hamburg, Germany

## Abstract

**Background:**

Preterm infants undergo multiple painful procedures, which may negatively affect neurodevelopment. Proper pain management, including opioid use, is essential. This study aimed to determine the impact of opioid administration in very and extremely preterm infants on cognitive, motor, and behavioral outcomes at the corrected age of 3 years.

**Methods:**

This retrospective, single-center study included preterm infants born between 23 and 32 weeks of gestation, admitted to the Medical University of Vienna between 2011 and 2017. Follow-up data were collected at 3 years corrected age. Primary outcomes included behavioral outcomes assessed by the Child Behavior Checklist (CBCL) and cognitive and motor outcomes using the Bayley Scales of Infant Development (BSID).

**Results:**

A total of 333 preterm infants were included, with 214 in the non-opioid group (no exposure to opioids) and 119 in the opioid-group (exposure to opioids). Significant differences in cognitive and motor scores were observed between the groups (92.5 (85.5–98.5) vs 88 (79–94) and 85 (76–96) vs 76 (67–85), both *p* = 0.001). Behavioral outcomes were within the normal range in both groups, although higher depressive scores were noted in the opioid group.

**Conclusions:**

Cumulative opioid exposure in neonatal care may negatively impact cognitive and motor development but did not significantly affect overall behavioral outcomes.

**Impact:**

- Our findings suggest that cumulative opioid exposure in the NICU does not significantly influence overall behavioral problems at the age of 3 years. However, it poses a risk for altered cognitive and motor development.
- This study highlights the distinct effects of opioid exposure on motor development and cognitive outcomes, while offering a nuanced perspective on behavioral outcomes, filling gaps in understanding the long-term neurodevelopmental consequences in preterm infants.
- The findings emphasize the need for careful management of opioid administration in NICU settings, balancing pain relief with potential long-term neurodevelopmental risks, while also underscoring the role of confounding factors such as IVH in shaping developmental trajectories.

## Introduction

Premature birth, defined as delivery before 37 weeks of gestation, is a significant public health concern, owing to its associated risks and the long-term implications it has on child development.^1^ One of the critical challenges in the care of preterm infants is the management of pain, particularly in neonatal intensive care unit (NICU).^2^ In these settings, preterm infants often undergo multiple painful procedures, underscoring the necessity for effective pain management strategies.^3^ Opioids, due to their potent analgesic properties, are commonly used in the NICU for pain relief. However, the potential long-term neurodevelopmental impact of opioid exposure in this vulnerable population remains a topic of considerable concern and ongoing research.^4–12^

The developing brain of preterm infants is especially vulnerable to external influences, making the management of pain and discomfort a delicate balance.^13,14^ While untreated pain can lead to negative neurodevelopmental outcomes, the use of pain-relieving medications, such as opioids, is not without risks. Studies have shown that exposure to opioids in early life can affect various aspects of neurological development, including cognitive and motor skills.^12,15–17^ However, a significant gap remains in our understanding of how these drugs impact behavioral performance, especially in the long term.

This study aims to shed light on this complex topic by examining the cognitive, motor, and behavioral performance of children born preterm at the corrected age of 3 years, focusing on those who were exposed to opioids during their NICU stay. By analyzing behavioral outcomes at this critical developmental stage, the study seeks to provide valuable insights into the long-term effects of neonatal opioid exposure.

## Methods

### Study design and patient population

This retrospective study was conducted at the Division of Neonatology and Pediatric Intensive Care of the Medical University of Vienna. The study focused on analyzing data pertaining to preterm infants admitted to our units S. The patient cohort was divided into two groups based on postnatal opioid exposure: the opioid group (received opioids) and the non-opioid group (did not receive any opioids). Approval for the study was obtained from the Ethics Committee of the Medical University of Vienna (EK-Code 1041/2021).

### Inclusion criteria

The study included very and extremely preterm infants born between 23 and 32 weeks of gestational age, hospitalized in the NICU of the Medical University of Vienna from January 1, 2011, to December 31, 2017.

### Exclusion criteria

Exclusion criteria included missing data from the follow-up clinic, as well as children born with congenital malformations and chromosomal aberrations. Missing data were handled using a complete case analysis approach, including only participants with fully documented main outcome variables, as the extent of missing data was minimal and did not require imputation. Additionally, all infants who received dexmedetomidine or clonidine were excluded from the analysis.

### Cumulative opioid dosage

Following the Vienna Protocol for Neonatal Pain Sedation (V-PNPS), infants considered in pain were treated with intravenous bolus and/or continuous morphine or fentanyl infusions during the NICU stay. To summarize the use of both drugs in one cumulative dose, a morphine equivalent dose was calculated using the following formula: morphine equivalent dosage = fentanyl dose × 100.^18^ Normalized data were derived by dividing the calculated cumulative opioid dosage (morphine and/or fentanyl) by the total number of treatment days. Opioids were only administered intravenously during the study period. No oral administration of opioids was documented.

### Three-year follow-up assessment

Cognitive, motor, and behavioral assessments were conducted at the corrected age of 3 years in our follow-up clinic. The Child Behavior Checklist (CBCL) was employed to evaluate behavior at the corrected age of 3 years.^19^ In addition, cognition and motor outcomes were assessed using the Bayley Scales of Infant Development (BSID), Second and Third Edition.^20^ Growth parameters, including weight, height, and head circumference, were also recorded.

### Child Behavior Checklist (CBCL)

The CBCL is a parent-reported questionnaire assessing behavioral problems in children and adolescents. The evaluation was based on the DSM-5-oriented scales, which were utilized for this study.^19^

### Bayley Scale of Infant Development (BSID)

The BSID is a widely used instrument for assessing cognitive and motor development in infants. As we used two different version of the scale (version II and III), scaled scores were calculated for each subtest and then converted into composite scores for cognitive and motor development.^20^ We therefore refer further in the manuscript to cognitive and motor development.

### Data extraction

Patient information was sourced from various computer systems, including iSmartView®, CareView®, ICIP® (IntelliVue Clinical Information Portfolio), catoPAN® (Cato Software Solutions), and AKIM® (Allgemeines Krankenhaus Informations-Management). Excel® (Microsoft Office 16 version) and IBM SPSS Statistics® (IBM Corporation, Armonk, NY) were used for storing and analyzing the database.

### Statistical analysis

For metric parameters following a normal distribution, the mean ± SD was calculated and illustrated using boxplots. For metric parameters that do not follow a normal distribution, the median (IQR) was calculated. For nominal (categorical) parameters, both absolute frequencies and percentages were specified.

A t-test was used to compare means (two-sided significance level of α = 0.05). A *χ*^2^ was used to compare categorical data. A Mann–Whitney *U*-test was performed to compare medians of variables that do not follow a normal distribution.

Multiple linear regression was performed to control for confounding factors and to analyze risk factors predictive of BSID and CBCL scores at the corrected age of 3 years. The regression model incorporated adjustments for the following risk factors: sex, gestational age, birth weight, cumulative opioid exposure, midazolam exposure, intraventricular hemorrhage (IVH) grade ≥ 3/4, severe retinopathy of prematurity (ROP > 2), necrotizing enterocolitis (NEC) requiring surgery, bronchopulmonary dysplasia (BPD), sepsis, and maternal education. Maternal education was used as a surrogate for socioeconomic status, as it is a well-established proxy for socio-economic factors that can impact child development, as demonstrated by Patra and colleagues.^21^ According to the Austrian school system, a cutoff was chosen to distinguish basic education from advanced education. Basic education includes levels from no formal education to completion of general/middle school without a final examination. Advanced education comprises achievements like attendance at vocational secondary school, completion of grammar/vocational school with a leaving examination, university education. This cutoff effectively distinguishes between general school education and more specialized or higher educational paths.

## Results

**Fig. 1:**
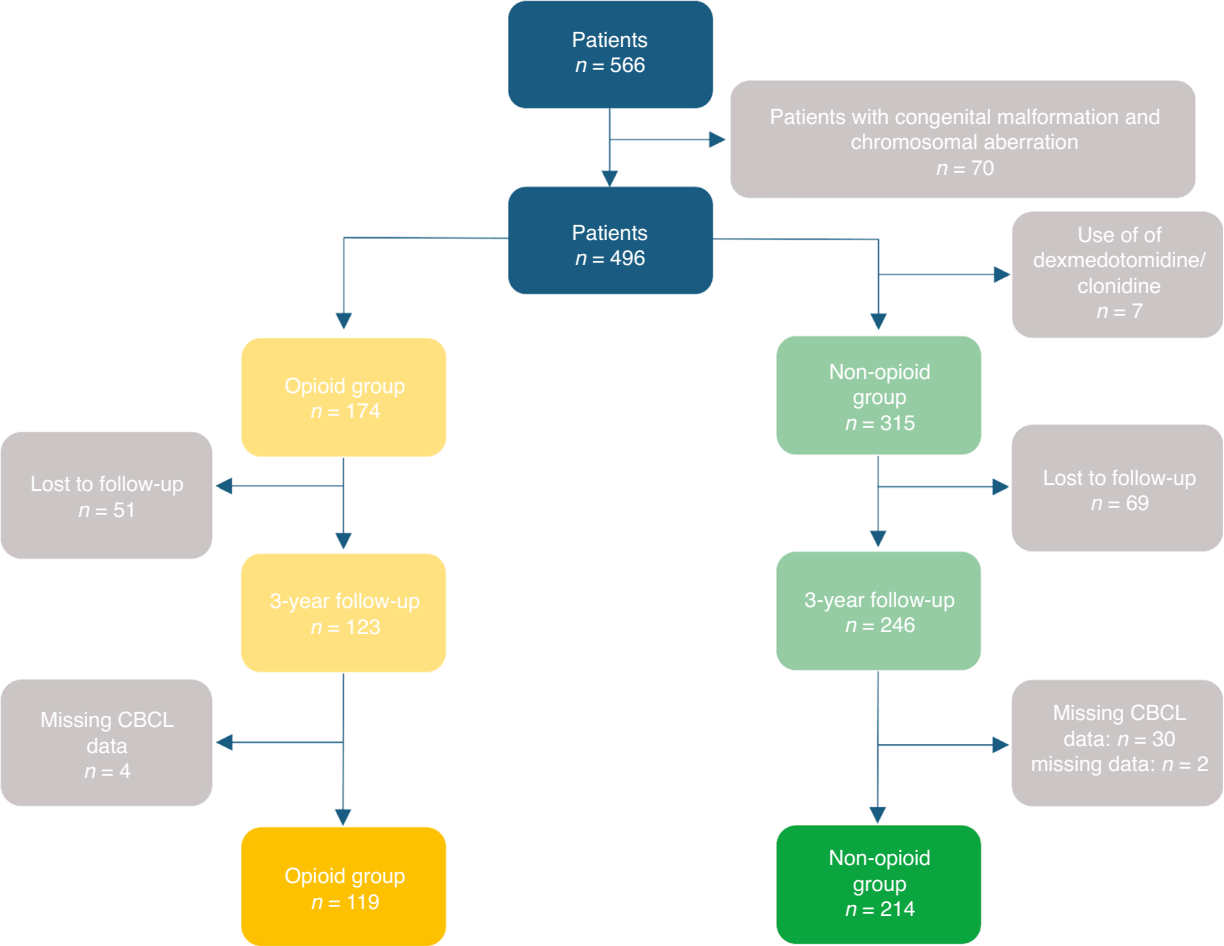
Overview of included and excluded patients.

A total of 333 patients were included in the analysis of this study. After applying the previously mentioned exclusion criteria, 119 patients were included in the opioid-group, compared to 214 patients in the non-opioid group. Figure 1 provides an overview of the excluded infants, as well as the total number of patients in both the opioid and non-opioid groups

### Descriptive characteristics

Baseline patient characteristics, intensive care therapy, in-hospital outcomes, and follow-up information are shown in Table 1. There were significant differences between the opioid and the non-opioid group regarding baseline characteristics. In the non-opioid group, birth weight, head circumference, and gestational age were significantly higher compared to the opioid group [954 (800–1155) vs 780 (605–962) grams, 26 (24–27) vs 24 (22–26) cm, 27 (26–28) vs 25 (24–28) weeks, respectively].

**Table 1:** Baseline Characteristics.

	Opioid-group (*n* = 119)	Non-opioid- group (*n* = 214)	*p* value
Patient characteristics			
Birthweight, g, median (IQR)	780 (605–962)	954 (800–1155)	<0.001*
Head circumference (birth), cm, median (IQR)	24 (22–26)	26 (24–27)	<0.001*
Gestation, week, median (IQR)	25 (24–28)	27 (26–28)	<0.001*
Small for Gestational age, *n* (%)	23 (19.3)	29 (13.5)	0.28
Male sex, *n* (%)	73 (61.3)	108 (50.4)	0.06
Caesarean section, *n* (%)	105 (88.2)	191 (89.2)	0.348
Mothers with advanced educational certification, *n* (%)	84 (70.58)	156 (72.2)	0.044*
Intensive care therapy			
Patients treated with intravenous opioid, *n* (%)	119 (100)	-	**-**
Total cumulative opiate dose, mg/kg, median (IQR)	7.72 (1.6–44.6)	-	**-**
Patients treated with midazolam, *n* (%)	48 (40.3)	-	-
Total cumulative midazolam dose, mg/kg, median (IQR)	0.75 (0.28–7.96)	-	-
Mechanical ventilation, *n* (%)	118 (99.2)	13 (6.0)	<0.001*
Time on mechanical ventilation, days, median (IQR)	9 (4–19)	1 (1–2)	<0.001*
In-hospital outcomes			
Severe intraventricular hemorrhage (IVH 3/4), *n* (%)	29 (24.4)	21 (9.8)	<0.001*
Shunt surgery, *n* (%)	4 (3.3)	-	-
Severe retinopathy of prematurity (ROP > 2), *n* (%)	67 (56.3)	41 (19.1)	<0.001*
NEC requiring surgery, *n* (%)	19 (16.0)	-	-
Bronchopulmonary dysplasia (BPD), *n* (%)	39 (32.8)	24 (11.2)	<0.001*
Sepsis, *n* (%)	17 (14.3)	14 (6.5)	0.020*
Time to discharge, days, median (IQR)	98.5 (70–174)	63 (51–74)	0.015*
Follow-up at 3 years			
Weight, Kg, median (IQR)	13 (12–15)	14 (13–16)	0.002*
Length, cm, median (IQR)	95 (92–98)	96 (93–99)	0.002*
Head circumference (birth), cm, median (IQR)	48 (47–50)	49 (48–50)	<0.001*
Bayley cognitive scores, median (IQR)	88 (79–94)	92.5 (85.5–98.5)	<0.001*
Bayley motor scores, median (IQR)	76 (67–85)	85 (76–96)	<0.001*

Counts and percentages are written as *n* (%), and parameters are shown as mean ± SD. Percentages refer to the percentage based in the particular group. For continuous not normally distributed variables, *p* value was calculated *U*-test, and for categorical variables, *p* value was calculated using a chi-square test.

*IVH* intraventricular hemorrhage, *ROP* retinopathy of prematurity, *NEC* necrotizing enterocolitis, *BPD* Bronchopulmonary dysplasia.

*Statistically significant (*p* < 0.05).

Outcomes of intensive care therapy varied between the groups. As the non-opioid group consisted exclusively of infants who did not receive any opioids, cumulative opioid exposure was only documented in the opioid group, with a median of 7.72 (1.6–44.6) mg/kg. Both the incidence (118 vs. 13) and duration of mechanical ventilation (9 days vs. 1 day) were significantly higher in the opioid group (Table 1). Furthermore, significant differences among in-hospital outcomes were observed. In the opioid group, severe IVH, severe ROP, BPD, and sepsis, as well as total length of stay in the NICU were significantly higher than in the non-opioid group (Table 1).

### Three-year follow-up assessment

There were significant differences in descriptive characteristics between the non-opioid and opioid groups regarding weight, height, and head circumference (Table 1).

**Fig. 2:**
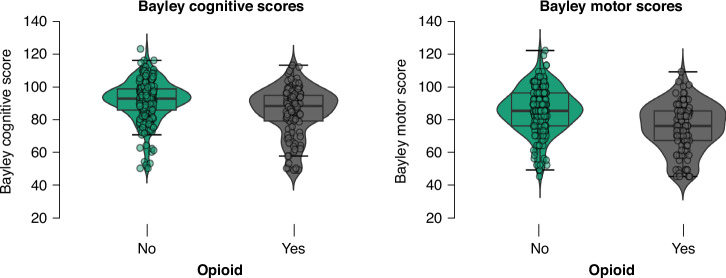
Violin plot of the mean scores distribution related to the cognitive and motor scores of the BSID.

When comparing means of BSID scores between the opioid and the non-opioid group at 3 years of age, significant differences were found in cognitive, as well as motor scores 88 (79–94) vs 92.5 (85.5–98.5) and 76 (67–85) vs 85 (76–96), both *p* < 0.001). (Table 1 and Fig. 2).

When looking at the CBCL, most of the included patients resulted to have behavioral scores within a normal range. Within the identified clinically relevant risk, autism was the most diagnosed (Supplementary Material, Likert Plot). This was true for the entire collective (Supplementary Material, Likert Plot) as well as for the non-opioid and opioid groups.

**Fig. 3:**
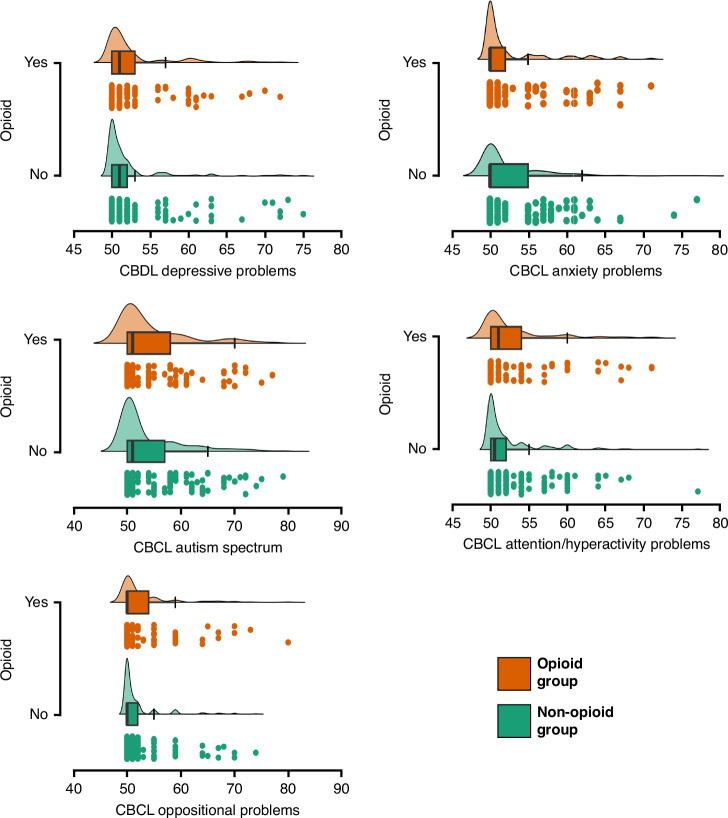
Mean scoring distribution of CBCL values based on DSM-V interpretation.

In fact, 8% of all patients (12 in the opioid group and 13 in the non-opioid group) were identified to be at risk for autism spectrum problems, followed by depressive problems (3%), Attention problems (2%), and oppositional defiant problems (2%). The distribution of clinically relevant scores was higher for autism spectrum in the opioid group (10% vs 6%) groups (Supplementary Material Likert Plot), but similar for other behavioral problems. The mean scores distribution for the clinically relevant CBCL categories and for both groups are plotted in Fig. 3, while Table 2 provides a median comparison of all CBCL variables considered. The unadjusted median analysis, using rank biserial correlation to determine effect size, reveals a median difference in raw scores for depressive problems in the opioid group, even within a similar score distribution (*p* = 0.034) (Table 2).

**Table 2:** CBCL differences between the opioid group and non-opioid group.

					95% CI for rank-biserial correlation	
	Opioid group	Non-opioid-group	*p* value	Rank-biserial correlation	Lower	Upper
Depressive problems (median, IQR)	51 (50–53)	51 (50–52)	0.034*	−0.133	−0.257	−0.004
Anxiety problems (median, IQR)	50 (50–52)	50 (50–55)	0.271	−0.065	−0.192	0.065
Autism spectrum problems (median, IQR)	51 (50–58)	51 (50–57)	0.245	−0.074	−0.201	0.056
Attention deficit/hyperactivity (median, IQR)	51 (50–54)	50.5 (50–52)	0.199	−0.080	−0.207	0.050
Oppositional defiant problems (median, IQR)	50 (50–54)	50 (50–52)	0.210	−0.075	−0.202	0.055

*Mann–Whitney test, effect size is given by the rank biserial correlation.

### Regression analysis for confounding factors

**Fig. 4:**
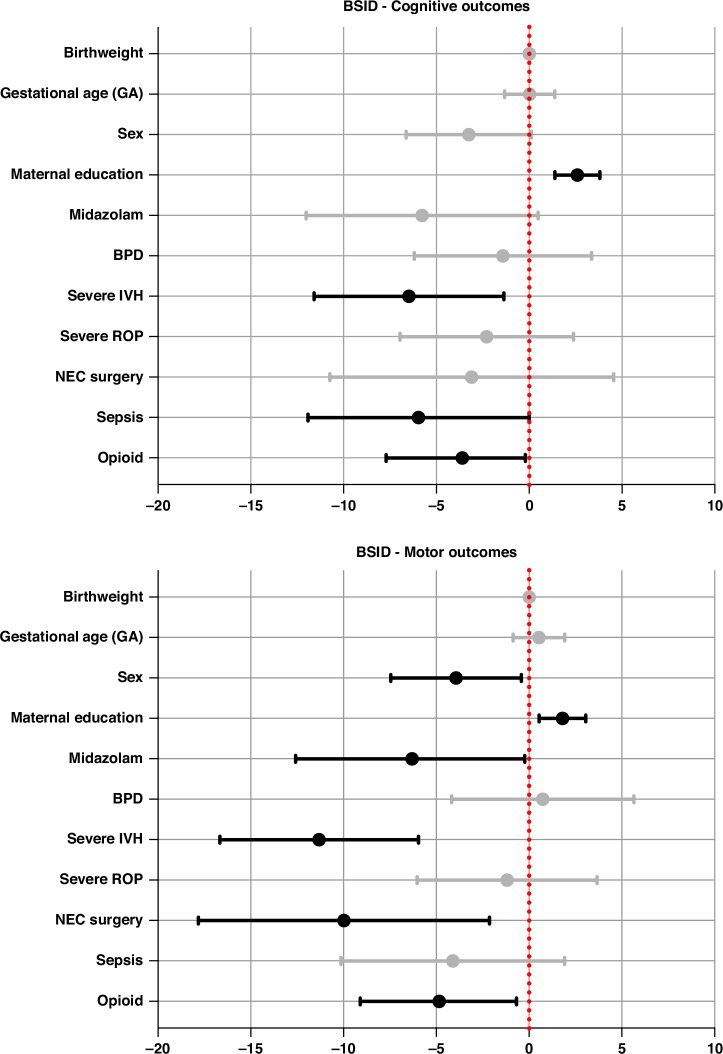
Linear regression model considering the impact of important in-hospital condition on outcome. BSID Bayley Scale of Infant Development, BW birth weight, IVH intraventricular hemorrhage, ROP retinopathy of prematurity, NEC necrotizing enterocolitis, BPD bronchopulmonary dysplasia, GA gestational age.

Cumulative opiate exposure (*p* = 0.034), severe IVH (*p* = 0.013), and sepsis (*p* = 0.05) were predictive for lower BSID cognitive scores, whereas higher education of the mother was predictive for higher BSID cognitive scores (*p* < 0.001) (Fig. 4, Supplementary Material, Regression). Furthermore, cumulative opioid exposure, male sex, severe IVH, midazolam, and NEC surgery were predictive for lower BSID motor scores (*p* = 0.026, *p* = 0.029, *p* = <0.001, *p* = 0.042, *p* = 0.013), whereas higher education of the mother was predictive for higher scores (*p* = <0.001) (Fig. 4, Supplementary Material, Regression).

**Fig. 5:**
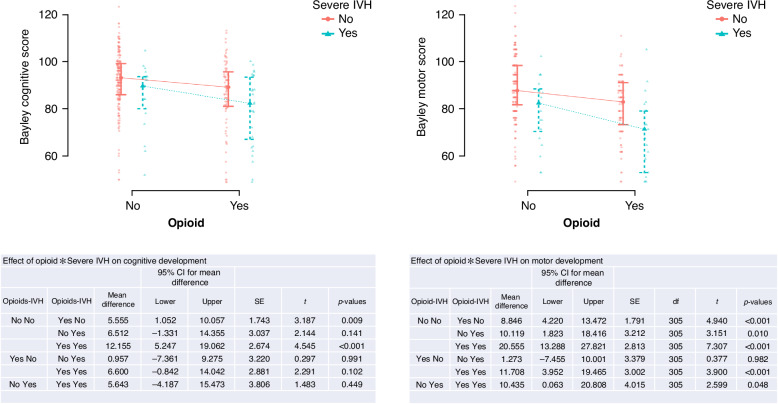
MANOVA contextualizing the impact of opioids and IVH on outcomes.

As severe IVH is a well-known condition having important consequences on outcomes, we have tried to further isolate the effect of IVH and opioids on outcomes by performing a MANOVA (Fig. 5; Supplementary Material, Descriptive Statistic). The MANOVA pointed out a significant effect for cognitive development on the interaction No opioids*No IVH vs. Yes opioids*Yes IVH (*p* = <0.001). Mean differences by comparing a group of patients without opioids and without IVH, with a group exposed to opioids only, or suffering severe IVH only, were similar (5.5 vs. 6.5). Both opioids and IVH showed a strong effect when present alone or together on motor development (*p* < 0.001).

**Fig. 6:**
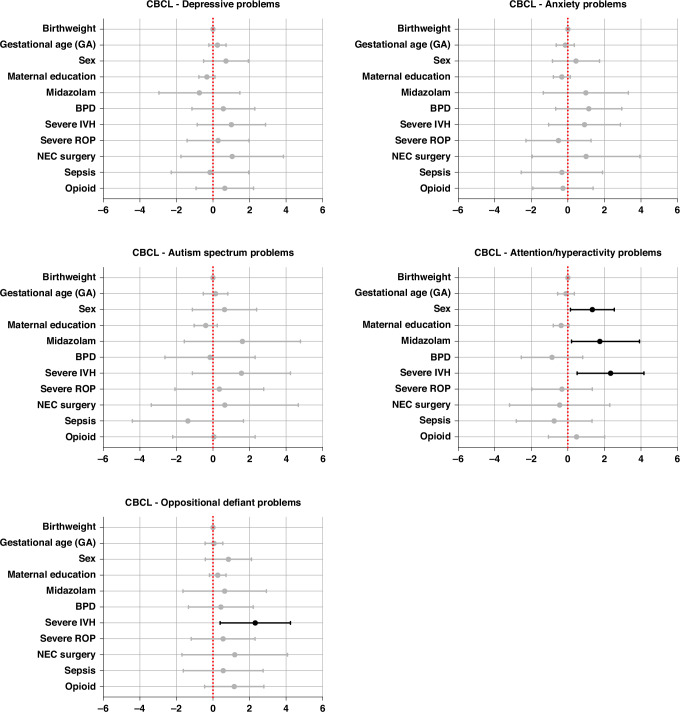
Linear regression model considering the impact of important in-hospital conditions on behavioral outcome. BSID Bayley Scale of Infant Development, BW birth weight, IVH intraventricular hemorrhage, ROP retinopathy of prematurity, NEC necrotizing enterocolitis, BPD bronchopulmonary dysplasia, GA gestational age.

When looking at the CBCL (Fig. 6), by controlling for other important factors, infants with severe IVH had a significantly higher risk for higher oppositional scores on the CBCL (*p* = .025). Severe IVH (*p* = .012), male sex (*p* = .029), and midazolam exposure (*p* = .050) were also associated with significantly higher risks for attention and hyperactivity problems (Fig. 6).

To better understand the impact of the level of opioid exposure on neurobehavioral outcomes, we also divided the opioid group according to the median of cumulative opioid exposure, which was defined to be 7.72 mg/Kg. We also compared this information with the group of patients not exposed to opioids. This provided us with the opportunity to perform a sub-analysis between two groups of opioid exposure (below the median vs. above the median). Yet, when looking at the BSID, no differences could be found in cognitive development for the two levels of opioid exposure groups (*p* = 0.129); however, the highest opioid exposure group and the non-opioid group showed significant differences (<0.001); finally, no difference were found between the lower opioids´ exposure group and the non-opioid exposure group (*p* = 0.128) (Supplementary Material, median opioid exposure sub-analysis). The same was not true for the motor outcomes, which revealed differences between all considered groups non-opioid group vs. above median group, *p* = <0.001; non-opioid group vs below the median group, *p* = 0.009; above median group vs below median group, *p* = 0.005 (Supplementary Material, median opioid exposure sub-analysis). Moreover, a significant difference was found in the attention/hyperactivity scale of the CBCL (*p* = 0.0397) when comparing patients with opioid exposure below and above the median to those without opioid exposure. (Supplementary Material, median opioid exposure sub-analysis).

## Discussion

In this retrospective study, differences between the groups (unadjusted values) could be found in both cognitive and motor scores. Behavioral scores based on DSM-5-oriented scales were mostly within the norms in both groups. However, increased scores (unadjusted values), but mostly within a normal range, were identified for depressive problems. Clinically relevant scores were higher for the Autism spectrum problems, showing an incidence of 8% for the entire collective. When comparing only patients below vs above the median opioids´ exposure, differences could be found in cognitive (non-opioid group vs above median group), motor (all interaction), and behavioral (attention problems) outcomes. Finally, the regression analysis identified a notable impact of opioid exposure as risk for cognitive and motor outcomes, as measured by the BSID. In addition, midazolam exposure may also be a risk factor for worse motor outcomes. While there was no impact of opioid exposure on behavioral outcomes at the age of 3 years, midazolam exposure may be a risk factor for attention and hyperactivity problems.

### Effects of opioids on cognitive outcomes

We observed a significant effect of opioid exposure during the postnatal NICU stay on cognitive scores at 3 years of age. When linear regression was used to account for risk factors, opioid exposure, sepsis, lower maternal education, and severe IVH were significant predictors for worse cognitive outcomes. A sub-analysis oriented to precisely understand the role of opioids and IVH on neurodevelopmental outcomes showed a similar effect of the isolated effect of opioids and IVH on outcomes, which was stronger when the two conditions were simultaneously considered. This was true for both cognitive and motor development, whereby the strongest effect was shown for motor outcomes. These findings align with the NEOPAIN trial, where continuous morphine doses were associated with altered response latencies at 5 to 7 years of age.^22^ Similarly, recent studies have linked postnatal morphine exposure to neurobehavioral problems and early alterations in cerebral structure, although these effects did not persist into childhood.^23^ Another study found that a higher cumulative opioid dose was associated with worse cognitive scores at 20 months corrected age.^24^

Our study aligns with the EPIPAGE-2 findings by demonstrating that opioid exposure is associated with poorer cognitive outcomes, reinforcing concerns about its long-term impact on neurodevelopment. Additionally, while EPIPAGE-2 primarily linked prolonged opioid and midazolam exposure to lower FSIQ, our study further highlights midazolam as a specific risk factor for poorer motor outcomes.^25^

Compared to our group’s earlier research, we observed mixed findings regarding the effects of cumulative opioid doses on neurodevelopmental outcomes. Specifically, two previous studies on neurodevelopmental outcomes revealed that increased opioid exposure under the post-implementation phase of the pain and sedation management protocol (V-PNPS) did not significantly affect cognitive outcomes in preschool-aged children.^9,11^ Nonetheless, it is important to mention that these studies compared an intervention group with heightened opioid exposure to a retrospective control group from the preceding year, prior to the V-PNPS implementation, where both groups were exposed to different levels opioids. In the present study, including a brighter collective of patients, a difference in cognitive development was notable when comparing group of patients exposed to opioids vs those who were not, but the same was not true when comparing patients below vs above the median cumulative opiods´ exposure.

### Effects of opioids on motor outcomes

This study revealed differences in BSID motor scores at 3 years of age among preterm infants exposed to opioids. After adjusting for risk factors using linear regression analysis, several risk factors, including opioid exposure, midazolam exposure, severe IVH, NEC surgery, lower maternal education, and male sex, were associated with adverse motor outcomes.

These findings are consistent with the NEOPAIN trial, where morphine was associated with overall lower motor scores.^26^ A more recent study by Puia-Dumitrescu and colleagues also observed poorer neurodevelopmental outcomes at 2 years of age following opioid exposure. In the mentioned study,^27^, considering a slightly larger sample size compared to ours, the authors found significant differences between the group exposed vs not exposed to opioids. Moreover, when they tried to differentiate the exposure to opioids, using a cut-off of more or less than 7 days of exposure, they found differences in BSID scores, and this was particularly true for the motor scores.

Our results also align with the findings of Puia-Dumitrescu et al. ^27^ who reported that exposure to both opioids and benzodiazepines in extremely preterm infants was associated with lower cognitive, motor, and language scores at 2 years of age.

This finding would be in line with ours, where motor scores remained significantly worse in the opioids group even after correcting for other important medical conditions or by performing sub analysis regarding the level of opioid exposure.

A recent meta-analysis including 36 studies on the impact of pain and opioids on rodents neurobehavioral outcomes, found no association between opioids and altered motor outcomes.^12^ However, the effects of pain and painful events were associated with neuronal cell death, anxiety, and depressive behavior.^12^ In another study, it was found that there was an inverse relationship between the number of invasive procedures and motor performance at 18 months.^28^ This finding aligns with prior observations indicating that skin-breaking procedures and untreated pain, rather than opioid exposure, are linked to altered motor outcomes.^28,29^

### Effects of opioids on behavioral outcomes

Our study indicated no significant effect of opioid exposure on overall behavioral outcomes, as measured by the CBCL, suggesting that cumulative opioid exposure did not noticeably impact overall behavioral outcomes at 3 years of age. This is in contrast with previous research by Steinhorn et al., revealing that preterm infants exposed to morphine exhibited more dysregulated behavior at 2 years of age.^23^ Contradictory results were noted in other studies, emphasizing that the potential effects of morphine on internalizing behaviors may become more evident as children grow older.^30,31^ In addition, our research group found a dose-dependent association of cumulative opioid exposure and abnormal scores on autism spectrum problem scales in the CBCL.^11^ Thus, a higher dosage of morphine administered in the postnatal period was associated with a clinically apparent impact on behavior. We could not replicate this data; however, even considering most of the CBCL scores were within the norm, risk of Autism spectrum problems was the most rated CBCL outcome in our sample. The incidence of risk of Autism spectrum problems in our population was 7.4%, which is in line with what has been described in a recent meta-analysis including 3366 infants.^32^

Furthermore, study identified midazolam exposure as a potential risk factor for oppositional defiant problems. This is in line with previous studies indicating that midazolam may have lasting effects, particularly on emotional and behavioral regulation.^33^ Preclinical models have shown that early benzodiazepine exposure, such as midazolam, can lead to neuroapoptosis and suppressed neurogenesis, disrupting critical brain development.^34,35^ Findings from a clinical study involving 140 preterm infants show that early exposure to midazolam is associated with impaired hippocampal growth, offering a potential explanation for the observed behavioral deficits.^36^ Since the hippocampus is critical for memory, learning, and emotional regulation, any disruption in its development could have profound effects on memory, learning, and emotional regulation.

#### Strengths and limitations

One of the key strengths of our study is the use of a standardized neonatal pain and sedation protocol, implemented in 2010,^37^ which ensured consistent management of pain and agitation across all participants and minimized variability in treatment practices throughout the study period. However, we are aware that due to the retrospective design and high turnover in our NICU, some deviations from the protocol may have occurred.

This study is limited by its retrospective, single-center nature. A notable limitation of our study was the diminishing size of the study population over time due to loss to follow-up and the application of other exclusion criteria. However, our dropout rate was in line with the dropout rates reported in the literature.^38^ Another limitation of this study that may have affected the measurement of behavioral outcomes was the presence of a greater number of confounding factors in the opioid group compared to the control group. Despite our efforts to account for the most significant risk factors, it was not possible to fully encompass the complete environmental context of our patients in the study. This limitation, however, is common among many clinical trials on this topic as usually more vulnerable preterm infants are sicker, require more intensive care, and therefore also more analgosedation.

In our study, we focused specifically on opioid exposure to better understand its impact on neurodevelopmental outcomes, and did not include other medications commonly used for pain and agitation management, such as clonidine, dexmedetomidine, or acetaminophen. While these medications are important in neonatal care, their inclusion would have expanded the scope of our study and was beyond the objectives of this analysis.

Despite these limitations, this study significantly contributes to the existing knowledge regarding the effects of opioids on cognitive, motor, and behavioral outcomes in preterm infants at 3 years of age. Additionally, it underscores the importance of considering risk factors such as severe IVH and environmental influences in this context.

## Conclusion

In our research, we observed that cumulative opioid exposure was significantly associated with lower cognitive and motor outcomes. Behavioral assessments using the CBCL did not show significant differences in various constructs after controlling for important medical conditions. The findings emphasize the need for careful opioid management in NICUs, balancing pain relief with long-term neurodevelopmental risks, while considering the impact of confounding factors like IVH on developmental outcomes.

## Supplementary information

Supplementary Material

## Data Availability

All data generated or analyzed during this study are included in this published article (and its supplementary information files). The datasets generated during and/or analyzed during the current study are available from the corresponding author upon reasonable request.
