# Supplementary material for: Cognitive, motor, and behavioral outcomes in preterm infants exposed to opioids
Source: Pediatr Res. 2025 Apr 12;98(3):918–27. doi: 10.1038/s41390-025-04048-3 (PMC12507652; doi:10.1038/s41390-025-04048-3)
Supplement: Supplementary file 1 — Supplementary Material [file 41390_2025_4048_MOESM1_ESM.pdf]

Descriptive Statistic Supplementary Material

Bayley Scales Descriptive Statistics

	Bayley_Mental_Score		Bayley_Motor_Score	
Opioid	No	Yes	No	Yes
Median	92.500	88.000	85.000	76.000
Mean	91.402	84.721	85.332	74.726
Std. Deviation	12.328	14.934	13.640	14.701
95% CI Std. Dev. Upper	13.635	17.206	15.118	16.914
95% CI Std. Dev. Lower	11.251	13.194	12.427	13.002
Minimum	50.000	49.000	45.000	45.000
Maximum	123.000	113.000	122.000	109.000

Marginal Means - Opioid ✻ Severe_IVH on Mental Outcomes

			95% CI for Mean Difference		
Opioid	Severe_IVH	Marginal Mean	Lower	Upper	SE
No	No	91.940	90.028	93.852	0.972
Yes		86.386	83.538	89.233	1.447
No	Yes	85.429	79.768	91.089	2.877
Yes		79.786	74.884	84.688	2.491

Marginal Means - Opioid ✻ Severe_IVH on Motor Outcomes

			95% CI for Mean Difference		
Opioid	Severe_IVH	Marginal Mean	Lower	Upper	SE
No	No	86.369	84.351	88.388	1.026
Yes		77.523	74.635	80.411	1.468
No	Yes	76.250	70.261	82.239	3.043
Yes		65.815	60.661	70.969	2.619

Impact of Cumulative Opioid Exposure (Above Median Exposure Vs Below Median Exposure)

	t	df	p	Mean Difference	SE Difference	Cohen's d	SE Cohen's d
Bayley_Mental_Score	1.753	107	0.083	4.883	2.786	0.336	0.194
Bayley_Motor_Score	3.086	109	0.003	8.217	2.662	0.586	0.198

Group Descriptives

	Group	Mean	SD	SE	Coefficient of variation
Bayley_Mental_Score	Below median	87.481	13.023	1.789	0.149
	Above median	82.598	15.839	2.117	0.192
Bayley_Motor_Score	Below median	79.127	12.450	1.679	0.157
	Above median	70.911	15.415	2.060	0.217

Likert Plots

Opioid No

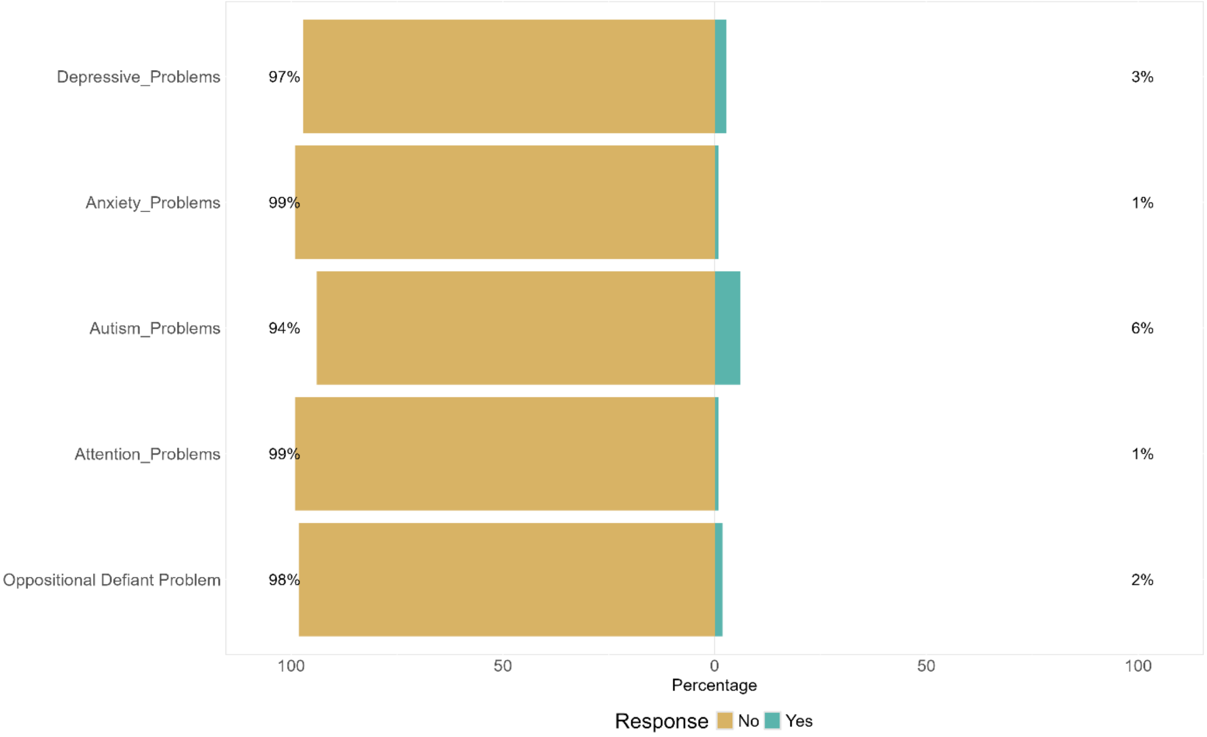

Opioid Yes

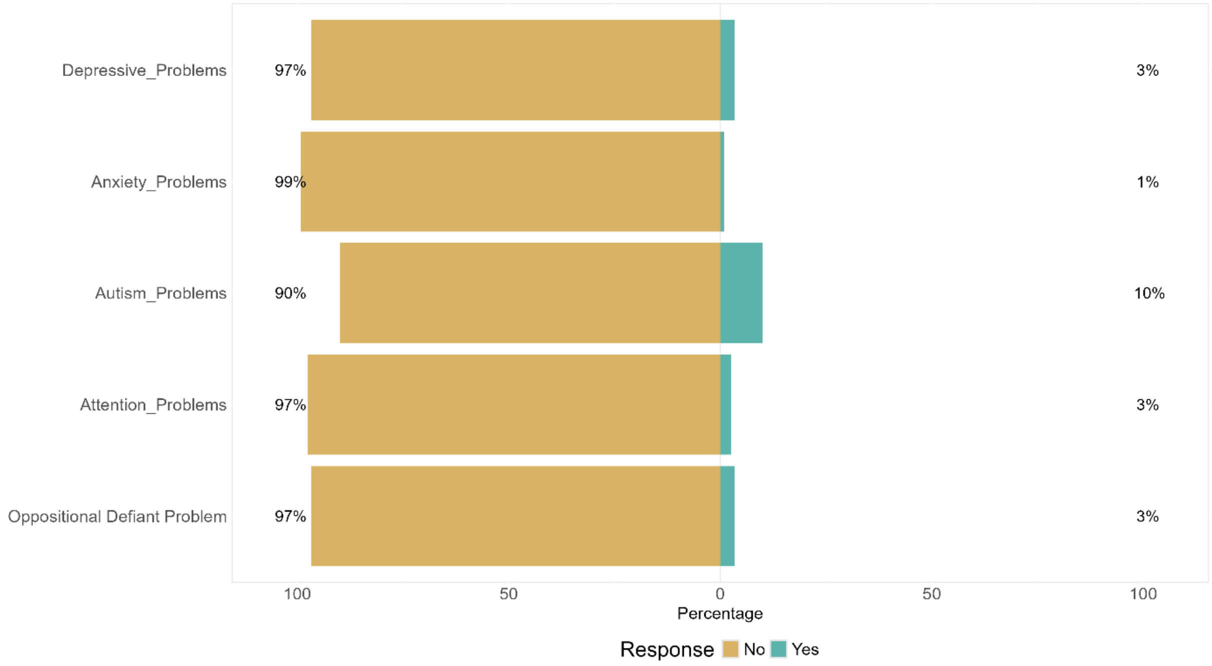

All Patients

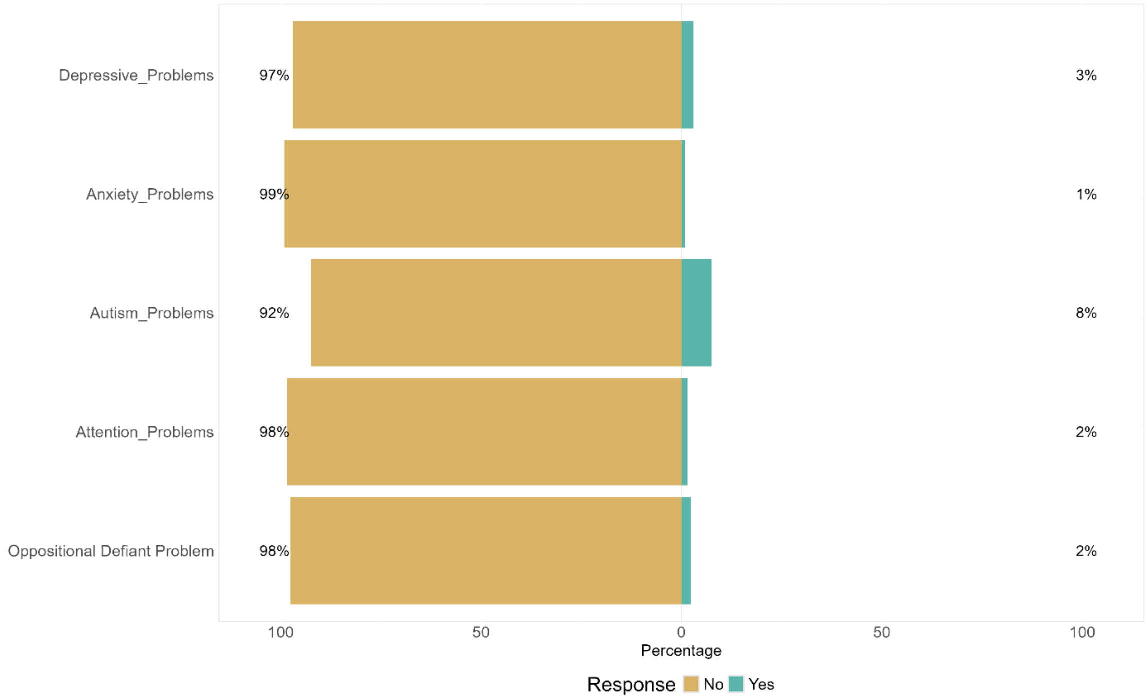

Regression Supplementary Material

BSID - Cognitive Outcomes									
Model		Unstandardized Coefficients		t	p	95,0% Confidence Interval		Collinearity Statistics	
		B	Std. Error			Lower Bound	Upper Bound	Tolerance	VIF
M1	(Constant)	81.787	17.083	4.788	<.001	48.099	115.475		
	Opioid	-3.603	2.083	-1.730	.034	-7.709	-.202	.640	1.563
	Sepsis	-5.964	3.020	-1.975	.050	-11.920	-.009	.948	1.055
	NEC Surgery	-3.095	3.877	-.798	.426	-10.740	4.550	.815	1.227
	Severe ROP	-2.291	2.369	-.967	.335	-6.964	2.381	.579	1.728
	Severe IVH	-6.476	2.590	-2.501	.013	-11.583	-1.369	.866	1.155
	BPD	-1.414	2.424	-.583	.560	-6.194	3.366	.834	1.199
	Midazolam	-5.770	3.171	-1.820	.070	-12.023	.482	.675	1.482
	Maternal Education	2.593	.614	4.226	<.001	1.383	3.803	.945	1.059
	Sex	-3.256	1.708	-1.907	.058	-6.623	.111	.977	1.023
	Gestational age (GA)	.022	.684	.032	.974	-1.328	1.372	.391	2.557
	Birthweight	.004	.005	.733	.464	-.006	.013	.439	2.280
a. Dependent Variable: Bayley mental at 3 years -Score									

BSID - Motor Outcomes

Model		Unstandardized Coefficients				95,0% Confidence Interval		Collinearity Statistics	
		B	Std. Error	t	p	Lower Bound	Upper Bound	Tolerance	VIF
M1	(Constant)	65.721	17.654	3.723	<.001	30.904	100.539		
	Opiates	-4.839	2.161	-2.239	.026	-9.101	-.677	.631	1.586
	Sepsis	-4.111	3.058	-1.345	.180	-10.141	1.919	.943	1.060
	NEC Surgery	-9.976	3.979	-2.507	.013	-17.823	-2.129	.826	1.211
	Severe ROP	-1.185	2.457	-.482	.630	-6.031	3.661	.570	1.756
	Severe IVH	-11.318	2.713	-4.172	<.001	-16.669	-5.968	.868	1.152
	BPD	.734	2.490	.295	.768	-4.176	5.645	.846	1.183
	Midazolam	-6.309	3.183	-1.982	.042	-12.585	-.232	.670	1.493
	Maternal Education	1.800	.636	2.831	.005	.546	3.053	.948	1.055
	Sex	-3.936	1.785	-2.205	.029	-7.456	-.416	.968	1.033
	Gestational age (GA)	.532	.705	.755	.451	-.858	1.923	.401	2.494
	Birthweight	.004	.005	.728	.467	-.006	.013	.436	2.294
a. Dependent Variable: Bayley motor at 3 years - Score									

CBCL – Depressive Problems

Model		Unstandardized Coefficients		t	Sig.	95,0% Confidence Interval		Collinearity Statistics	
		B	Std. Error			Lower Bound	Upper Bound	Tolerance	VIF
	(Constant)	46.491	5.959	7.802	<.001	34.744	58.239		
	Opioid	.646	.797	.811	.418	-.925	2.218	.637	1.570
	Sepsis	-.165	1.075	-.153	.878	-2.284	1.954	.935	1.070
	NEC Surgery	1.052	1.422	.740	.460	-1.751	3.855	.829	1.206
	Severe ROP	.280	.859	.325	.745	-1.415	1.974	.575	1.738
	Severe IVH	1.008	.948	1.063	.289	-.862	2.878	.857	1.167
	BPD	.567	.872	.650	.517	-1.153	2.287	.841	1.189
	Midazolam	-.743	1.124	-.662	.509	-2.958	1.472	.665	1.503
	Education Mother	-.333	.223	-1.492	.137	-.773	.107	.956	1.046
	Sex	.711	.622	1.143	.254	-.516	1.937	.974	1.027
	Gestational age (GA)	.244	.238	1.027	.306	-.225	.714	.434	2.305
	Birthweight	-.001	.002	-.317	.752	-.004	.003	.458	2.183

a. Dependent Variable: CBCL Data at 3 years - depressive problems

CBCL – Anxiety Problems

Model		Unstandardized Coefficients		t	Sig.	95,0% Confidence Interval for B		Collinearity Statistics	
		B	Std. Error			Lower Bound	Upper Bound	Tolerance	VIF
	(Constant)	56.278	6.262	8.987	<.001	43.932	68.625		
	Opioid	-.266	.838	-.317	.751	-1.917	1.386	.637	1.570
	Sepsis	-.324	1.130	-.287	.775	-2.551	1.903	.935	1.070
	NEC Surgery	.997	1.494	.667	.505	-1.949	3.943	.829	1.206
	Severe ROP	-.513	.903	-.568	.571	-2.293	1.268	.575	1.738
	Severe IVH	.914	.997	.917	.360	-1.051	2.879	.857	1.167
	BPD	1.146	.917	1.250	.213	-.662	2.954	.841	1.189
	Midazolam	.986	1.181	.835	.405	-1.342	3.314	.665	1.503
	Education Mother	-.329	.235	-1.401	.163	-.791	.134	.956	1.046
	Sex	.447	.654	.684	.495	-.842	1.736	.974	1.027
	Gestational age (GA)	-.141	.250	-.563	.574	-.634	.352	.434	2.305
	Birthweight	.001	.002	.567	.572	-.003	.005	.458	2.183
a. Dependent Variable: CBCL Data at 3 years - anxiety problems									

CBCL – Autism Spectrum Problems

Model		Unstandardized Coefficients		t	Sig.	95,0% Confidence Interval		Collinearity Statistics	
		B	Std. Error			Lower Bound	Upper Bound	Tolerance	VIF
	(Constant)	52.078	8.550	6.091	<.001	35.221	68.936		
	Opioid	.050	1.144	.043	.965	-2.205	2.305	.637	1.570
	Sepsis	-1.372	1.542	-.889	.375	-4.412	1.669	.935	1.070
	NEC Surgery	.646	2.040	.317	.752	-3.376	4.668	.829	1.206
	Severe ROP	.347	1.233	.281	.779	-2.084	2.778	.575	1.738
	Severe IVH	1.557	1.361	1.144	.254	-1.126	4.240	.857	1.167
	BPD	-.159	1.252	-.127	.899	-2.627	2.309	.841	1.189
	Midazolam	1.610	1.612	.999	.319	-1.569	4.789	.665	1.503
	Education Mother	-.397	.320	-1.240	.216	-1.029	.234	.956	1.046
	Sex	.631	.893	.707	.480	-1.128	2.391	.974	1.027
	Gestational age (GA)	.141	.342	.413	.680	-.532	.814	.434	2.305
	Birthweight	-.001	.002	-.481	.631	-.006	.004	.458	2.183

a. Dependent Variable: CBCL Data at 3 years - autism spectrum problems

CBCL – Attention Deficit/Hyperactivity Problems

Model		Unstandardized Coefficients		t	Sig.	95,0% Confidence Interval		Collinearity Statistics	
		B	Std. Error			Lower Bound	Upper Bound	Tolerance	VIF
	(Constant)	53.632	5.827	9.204	<.001	42.144	65.120		
	Opioid	.479	.779	.615	.539	-1.057	2.016	.637	1.570
	Sepsis	-.750	1.051	-.713	.476	-2.822	1.323	.935	1.070
	NEC Surgery	-.445	1.390	-.320	.749	-3.186	2.296	.829	1.206
	Severe ROP	-.321	.840	-.382	.703	-1.977	1.336	.575	1.738
	Severe IVH	2.340	.927	2.523	.012	.512	4.169	.857	1.167
	BPD	-.864	.853	-1.012	.313	-2.545	.818	.841	1.189
	Midazolam	1.753	1.099	1.596	.05	0.202	3.920	.665	1.503
	Education Mother	-.366	.218	-1.675	.095	-.796	.065	.956	1.046
	Sex	1.342	.608	2.206	.029	.142	2.541	.974	1.027
	Gestational age (GA)	-.101	.233	-.433	.665	-.560	.358	.434	2.305
	Birthweight	.001	.002	.731	.466	-.002	.005	.458	2.183
a. Dependent Variable: CBCL Data at 3 years – attention deficit/hyperactivity problems									

CBCL – Oppositional Defiant Problems

Model		Unstandardized Coefficients		t	Sig.	95,0% Confidence Interval for B		Collinearity Statistics	
		B	Std. Error			Lower Bound	Upper Bound	Tolerance	VIF
	(Constant)	43.304	6.140	7.053	<.001	31.199	55.408		
	Opioid	1.164	.821	1.417	.158	-.456	2.783	.637	1.570
	Sepsis	.559	1.108	.505	.614	-1.624	2.743	.935	1.070
	NEC Surgery	1.191	1.465	.813	.417	-1.697	4.079	.829	1.206
	Severe ROP	.554	.885	.625	.533	-1.192	2.299	.575	1.738
	Severe IVH	2.314	.977	2.368	.019	.388	4.241	.857	1.167
	BPD	.433	.899	.481	.631	-1.340	2.205	.841	1.189
	Midazolam	.640	1.158	.553	.581	-1.643	2.922	.665	1.503
	Education Mother	.262	.230	1.138	.256	-.192	.715	.956	1.046
	Sex	.843	.641	1.315	.190	-.421	2.107	.974	1.027
	Gestational age (GA)	.055	.245	.224	.823	-.429	.538	.434	2.305
	Birthweight	.004	.002	2.291	.023	.001	.007	.458	2.183
a. Dependent Variable: CBCL Data at 3 years - oppositional defiant problems									

Median Opioid exposure Sub-analysis

Bayley Cognitive Score – Sub-analysis on median distribution

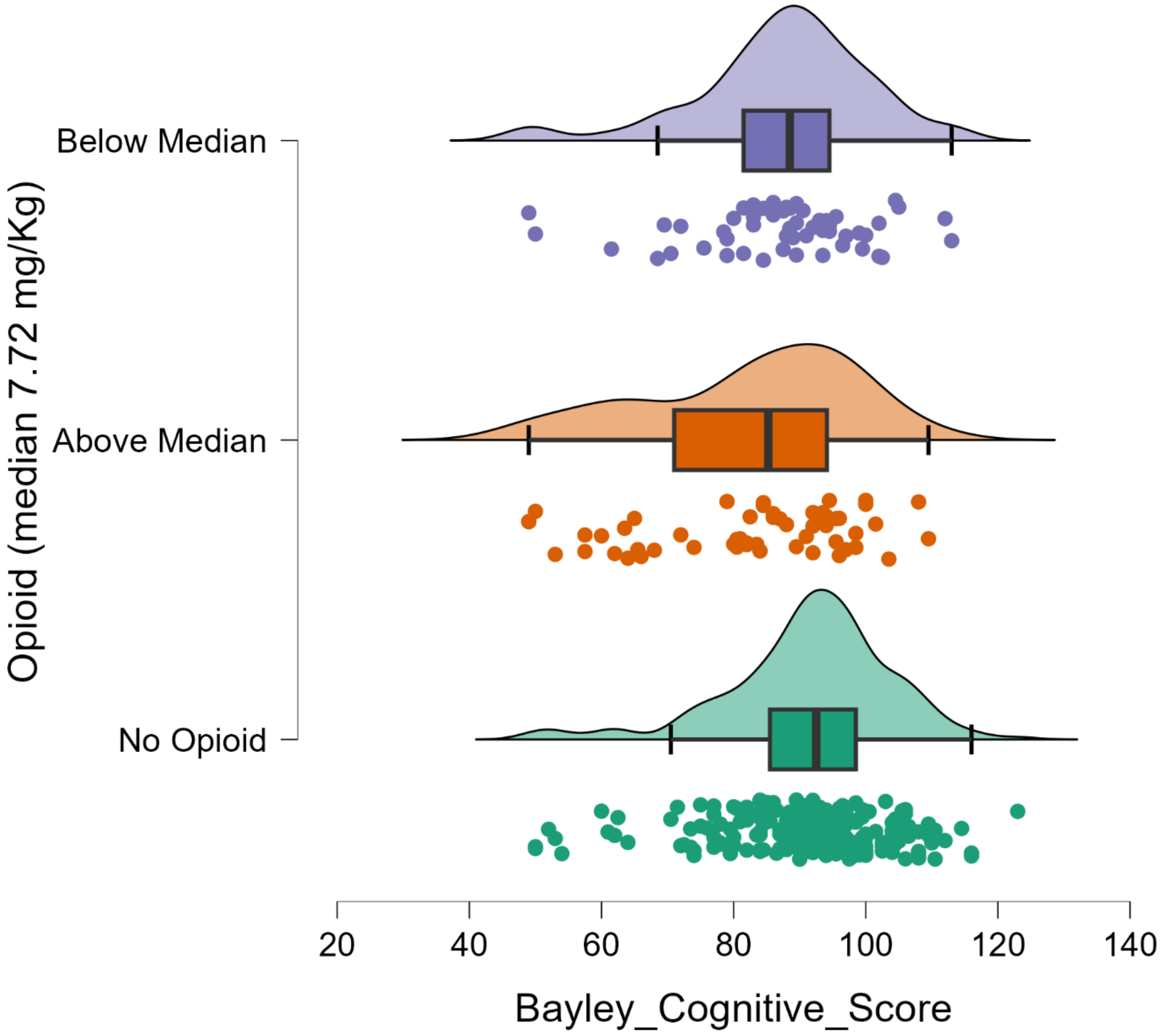

Cognitive Post Hoc Comparisons - Opioid Group

		Mean Difference	95% CI for Mean Difference		SE	df	t	p_{tukey}
			Lower	Upper				
No Opioid	Above Median	8.804	4.158	13.450	1.973	316	4.462	< .001
	Below Median	3.921	-0.827	8.670	2.017	316	1.945	0.128
Above Median	Below Median	-4.883	-10.803	1.037	2.514	316	-1.942	0.129

Bayley Motor Score– Sub-analysis on median distribution

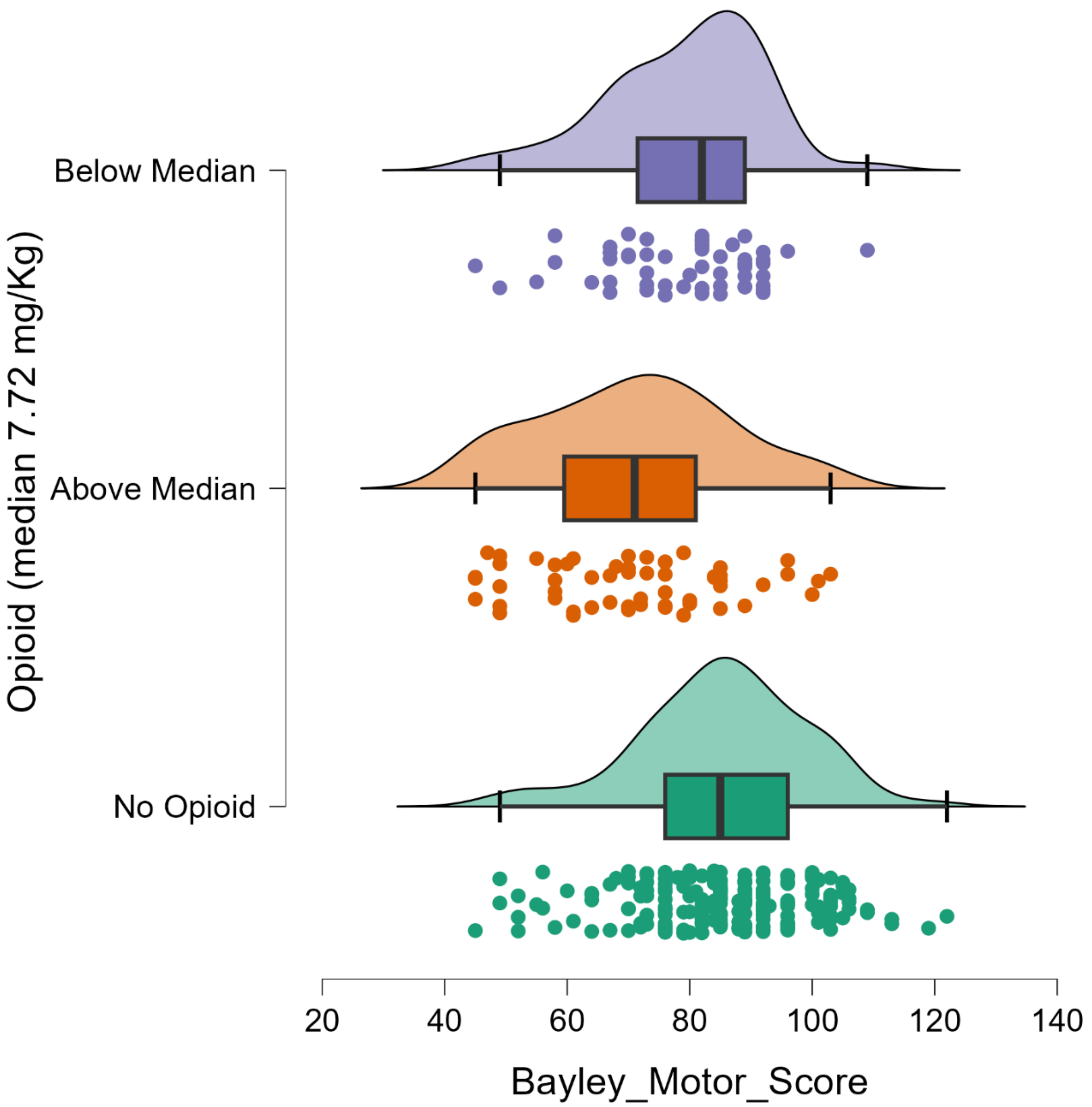

Motor Post Hoc Comparisons - Opioid Group

			95% CI for Mean Difference					
		Mean Difference	Lower	Upper	SE	df	t	p_{tukey}
No Opioid	Above Median	14.421	9.521	19.321	2.081	310	6.931	< .001
	Below Median	6.204	1.270	11.139	2.095	310	2.961	0.009
Above Median	Below Median	-8.217	-14.376	-2.057	2.615	310	-3.142	0.005

*Depression Problems: Post Hoc Comparisons - No Opioid*Above Median* Below Median*

		Mean Difference	95% CI for Mean Difference		SE	df	t	p_{tukey}
			Lower	Upper				
No Opioid	Above Median	-0.706	-2.285	0.873	0.671	327	-1.053	0.544
	Below Median	-0.637	-2.216	0.942	0.671	327	-0.950	0.609
Above Median	Below Median	0.069	-1.912	2.050	0.841	327	0.082	0.996

*Anxiety Problems: Post Hoc Comparisons - No Opioid*Above Median* Below Median*

		Mean Difference	95% CI for Mean Difference		SE	df	t	p_{tukey}
			Lower	Upper				
No Opioid	Above Median	0.089	-1.459	1.636	0.657	327	0.135	0.990
	Below Median	-0.377	-1.925	1.171	0.657	327	-0.573	0.834
Above Median	Below Median	-0.466	-2.407	1.476	0.825	327	-0.565	0.839

*Autism Spectrum: Post Hoc Comparisons - No Opioid*Above Median* Below Median*

		Mean Difference	95% CI for Mean Difference		SE	df	t	p_{tukey}
			Lower	Upper				
No Opioid	Above Median	-1.245	-3.403	0.912	0.916	327	-1.359	0.364
	Below Median	0.272	-1.886	2.429	0.916	327	0.297	0.953
Above Median	Below Median	1.517	-1.189	4.224	1.150	327	1.320	0.385

*Attention Problems: Post Hoc Comparisons - No Opioid*Above Median* Below Median*

		Mean Difference	95% CI for Mean Difference		SE	df	t	p_{tukey}
			Lower	Upper				
No Opioid	Above Median	-0.197	-1.695	1.300	0.636	327	-0.310	0.948
	Below Median	-1.559	-3.057	-0.062	0.636	327	-2.452	0.039
Above Median	Below Median	-1.362	-3.241	0.517	0.798	327	-1.707	0.204

*Oppositional Problems: Post Hoc Comparisons - No Opioid*Above Median* Below Median*

		Mean Difference	95% CI for Mean Difference		SE	df	t	p_{tukey}
			Lower	Upper				
No Opioid	Above Median	-0.794	-2.420	0.832	0.691	327	-1.149	0.484
	Below Median	-1.346	-2.972	0.281	0.691	327	-1.948	0.127
Above Median	Below Median	-0.552	-2.592	1.488	0.866	327	-0.637	0.800
